# Supplementary material for: Are carbon emissions trading and green financial instruments synergistic? -Comprehensive quantitative research based on content analysis
Source: PLoS One. 2024 Mar 7;19(3):e0298601. doi: 10.1371/journal.pone.0298601 (PMC10919601; doi:10.1371/journal.pone.0298601)
Supplement: S1 Data — (ZIP) [file pone.0298601.s001.zip › Data/地级市Z_F工作报告.zip!/┴╔─■/░░╔╜╩╨/└┤╫╘╣1⁄2╓┌║┼í╛┬φ┐╦╩2╛▌═°í┐_2012.docx]

各位代表：

现在，我代表市政府向大会报告工作，请予审议，并请市政协各位委员提出意见。

一、2011年政府工作回顾

刚刚过去的一年，在市委的正确领导下，在市人大、市政协的监督支持下，市政府深入贯彻落实科学发展观，带领全市人民团结奋斗，努力拼搏，圆满完成了市十四届人大四次会议确定的各项任务，实现了“十二五”良好开局。预计全年实现地区生产总值2444亿元，同比增长15%；地方财政一般预算收入208亿元，增长16%；全社会固定资产投资1400亿元，增长30%；城市居民人均可支配收入21223元，增长15.2%；农民人均纯收入11007元，增长19%。在国家统计局对全国282个地级市综合实力评比中,我市排名第24位。

（一）深入实施“工业五项工程”，工业经济实现较快增长

坚持工业立市，以重点发展主导产业集群推进工业优化升级，以重大工业项目建设积蓄发展后劲。全年新增规模以上企业100户。预计全年实现规模以上工业增加值1050亿元，增长15%。鞍山经济开发区成为国家新型工业化产业示范基地。

实施产业集群工程，主导产业支撑作用进一步增强。预计钢铁深加工产业集群实现销售收入1460亿元，增长16.3%；菱镁新材料产业集群实现销售收入645亿元，增长35%；装备制造产业集群实现销售收入494亿元，增长40%；化工新材料产业集群实现销售收入296亿元，增长47.5%；光伏光电产业集群实现销售收入33.5亿元，增长71.9%，五大主导产业销售收入占全市工业比重达到85%。

实施企业提升工程，骨干企业不断发展壮大。鼓励和支持企业通过“上船重组、上市融资、上档改造”做大做强。华冶与兵装天威集团、紫竹与中冶京诚公司、发蓝钢带与鞍钢实现重组。聚龙股份、森远股份和鞍重股份3家企业成功上市。鞍钢销售收入突破千亿元。全市销售收入超10亿元企业新增5户，达到22户。

实施项目工程，重大工业项目加快建设。全年开工建设投资千万元以上工业项目650个，总投资1054亿元。其中，投资亿元以上项目225个，投资10亿元以上项目20个。鞍钢投资57亿元的30万吨取向硅钢和投资10亿元的60万吨高档线材改造、宝得公司投资12亿元的150万吨H型钢、紫竹集团投资10亿元的80万吨船用钢构、后英集团投资10亿元的160万吨棒线材和投资10亿元的玻镁板、中钢集团投资10亿元的12万吨煤系针状焦等一大批重点项目竣工投产。

实施企业并购工程，科技创新能力不断提高。全年并购国内外科技型企业12户，引进海外研发团队13个，新增国家高新技术企业23家，高新技术产品增加值增长30%，我市被国家授予科技进步先进市。

实施淘汰落后工程，节能减排力度加大。淘汰落后产能企业9户，全年万元GDP能耗下降3.9%，工业增加值能耗降低6.5%，超额完成省下达任务。

（二）大力发展现代服务业，城区经济实力显著增强

调整行政区划，下放管理权限。超额完成第一轮城区经济“三年倍增”计划。预计城区财政一般预算收入完成36.6亿元，增长27.5%，高于全市平均增幅11.5个百分点。

服务业集聚区加快建设。重点推进11个市级以上服务业集聚区建设。汤岗子新城完成规划编制并启动实施，鞍南大道、建国大道、凤凰湖等基础设施开工建设,总投资1600多亿元的12个重大项目签约落地,港中旅温泉城等标志性龙头项目完成注资和土地摘牌。达道湾现代服务业集聚区、铁东中央商务集聚区、汤岗子温泉旅游休闲集聚区和海城西柳商贸集聚区被确定为省级服务业集聚区，汤岗子温泉旅游度假区被确定为辽宁省温泉旅游核心区，千山风景区被评为全国生态文化示范基地，我市被确定为国家现代服务业发展综合试点市。预计全年服务业增加值增长15.2%，社会消费品零售额增长17.7%。居民消费价格指数全年上涨4.5%，低于全国、全省平均水平。

金融业快速发展。预计金融机构本外币各项贷款余额1200亿元，增长16.3%；新增贷款180亿元，增长15.8%。鞍山银行跻身全国银行50强，兴业、光大等7家股份制商业银行陆续开业。新增小额贷款公司46家、村镇银行3家，引进基金公司4家。海城成功发行地方债券8亿元。

（三）全面推进“三化”，县域经济发展势头强劲

县域经济继续保持高速增长态势，预计完成地区生产总值1195亿元，增长22%，高于全市平均增幅7个百分点；财政一般预算收入55.3亿元，增长28%，高于全市平均增幅12个百分点。

农业产业化水平全面提升。“一县一业”取得新突破，海城大蒜种植发展到5万亩，成为国家现代农业示范县；台安鸭鹅饲养量达到1亿只，成为东北鸭鹅第一县；岫岩蘑菇产量达到60万吨，成为全国蘑菇第一县。全年新发展设施农业15万亩，总面积达到145万亩。实施亿元以上农产品深加工项目37个，其中6个投资10亿元以上项目开工建设。粮食总产量突破150万吨，创历史最高水平。

农村工业化进程不断加快。以县域工业园区为载体，推进工业产业集群建设，开工千万元以上工业项目403个。海城菱镁新材料、台安石油化工、岫岩矿产品深加工产业集群分别实现销售收入418亿元、50亿元和100亿元。

农村城镇化建设扎实推进。高标准建设和改造县城，全年县域房地产开发面积达到930万平方米，增长38.9%，继续走在全省前列。完成“四城一镇”基础设施投资120亿元，新城新市镇建设取得明显成效。

（四）全力推进对外开放，招商引资成效显著

对外招商成果丰硕。全年开展大型招商活动26次，引进了一批投资超亿元、10亿元乃至百亿元的大项目。共签约亿元以上项目289个，总投资3919亿元，其中10亿元以上项目83个，50亿元以上项目36个，100亿元以上项目11个。预计全年实际利用外商直接投资11亿美元，增长21.8%；引进域外资金820亿元，增长25%；出口创汇21亿美元，增长20%。

对上争取资金效果显著。用好用足国家政策，争取国家和省资金、政策支持，全年对上争取资金100亿元，比上年增长86%。

（五）大力推进基础设施建设和生态环境整治，城乡环境进一步改善

基础设施建设全面推进。完成千山东路改扩建主体工程、南三环和旧腾路续建工程，丹海高速公路建成通车,万水河南路、北路和鞍千路改造工程加快建设，新建农村公路100公里。陈家台桥、曙光路桥竣工通车。高铁鞍山西站和海城西站、鞍山客运北站和海城牛庄客运站竣工。新购置城市公交车100台。首山水源置换工程加速推进。新建、扩建6座大型集中供热锅炉房，更新改造管网33.5公里，新增供暖能力1千万平方米。

环境整治成效显著。开展市容市貌“十项综合整治”，取缔占道市场40处，拆除违章建筑594处，建筑施工噪声扰民等问题得到有效遏制。全面落实人大议案，建成11座垃圾中转站和2479个地埋式垃圾箱，城市垃圾实现定点定时清运。实施101个村庄连片综合整治示范工程，建成1万个农村无害化卫生厕所。整治万水河河道6公里，胜利湖、东山湖、杨柳湖形成水面景观。

生态建设全面加强。启动青山工程、蓝天工程、碧水工程。恢复矿山植被630亩，建设封山禁牧围栏49公里，清理小开荒1.5万亩，植树造林36万亩，新增城市绿地50万平方米，完成玉佛山森林公园勘界。鞍钢炼铁总厂和齐大山铁矿脱硫工程竣工，二烧车间脱硫项目开工建设，开展扬尘污染专项整治，城区89%以上天数的空气环境质量达到国家二级标准。积极推进判甲炉、大孤山、宁远等11座污水处理厂建设和运行，实施辽河污染防治、河道整治和生态建设工程，流域生态环境明显改善。

（六）积极保障和改善民生，社会建设进一步加强

重点民生工程全面推进。为城乡群众办的20件实事全部落实。提供大学生就业岗位5.4万个，军转干部全部安置，全年实现实名制就业12万人，城镇登记失业率1.97%。在全省率先建立高于国家标准的城镇居民社会养老保险制度，1.2万名“老工伤”人员纳入市级统筹管理。企业退休人员养老金待遇人均提高140元，城镇居民医疗保险和新农合政府补助均提高1倍以上，居民医保住院费用报销综合比例提高22.5%，城乡低保和失业金发放标准均提高10%以上。完成1.5万套保障性住房任务，192户城市单亲贫困母亲入住廉租房。建设回迁房项目22个，安置居民1万多户、3万余人。救助城乡贫困居民1.8万余人次，资助5万名困难家庭学生就学，对全市先天性心脏病、唇腭裂儿童和白内障老人免费实施手术500例。在全省率先成立食品药品犯罪侦查支队和大队，破获制假售假案件70起，打掉非法生产经营窝点30个。成功处置海城牛庄镇炭疽病疫情，实现疫情不扩散、患者无死亡。全市重特大安全事故为零，生产安全事故下降14.3%。严厉打击各种犯罪，人民群众安全感进一步增强。

社会事业全面进步。新建34所公立幼儿园和14所中小学校，9000余名进城务工人员子女免费接受九年义务教育，市职教城被确定为国家职业教育综合改革试点单位。完成医改三年试点任务，公立医院改革全面推进，组建中国医大鞍山医院等三大医疗集团，建设10余家各具特色的专科医院，全面实行基本药物零差率销售，公共卫生中心、肿瘤医院等项目加快建设。科技图书馆主体竣工，新建18个乡镇综合文化站，在全省率先完成农家书屋建设任务，提前3年完成“十二五”广播电视村村通工程。市奥体中心场馆主体竣工，新建100个健身广场，建成玉佛山健身长廊，鞍山籍运动员荣获7个世界冠军，为鞍山赢得了荣誉。人口和计划生育、人防和民兵预备役、法制、监察、民族、宗教、外事、侨务、对台、老龄、残联、妇女、儿童、统计、气象、档案、史志等各项工作都取得新成绩。

各位代表，过去的一年，是全市经济社会协调发展的一年，是招商引资上项目取得重大成果的一年，是城乡人民生活不断改善的一年。这些成绩，是在国家加强宏观调控和实施最严格土地管理的形势下取得的，是在钢铁行业不景气和房地产业下滑的不利形势下取得的，来之不易。这些成绩的取得，得益于市委的正确领导和市人大、市政协的大力支持，得益于鞍钢等中省直单位的重大贡献，得益于各县（市）区、各部门的辛勤工作，得益于全市广大干部的只争朝夕、埋头苦干，得益于全市人民的团结奋斗、努力拼搏。在此，我谨代表市政府向为鞍山发展付出艰辛的各位代表和委员，向奋战在全市各条战线的广大干部群众，向各民主党派、工商联、无党派人士、人民团体，向中省直单位、驻鞍部队、武警官兵，向关心支持鞍山发展的老领导、老同志，向所有为鞍山发展作出贡献的海内外朋友，表示衷心的感谢和崇高的敬意！

在肯定成绩的同时，我们也清醒地认识到，我市经济社会发展还存在许多矛盾和问题。主要是：经济结构调整任重道远，战略性和牵动性的重大工业项目少，新兴产业项目不多，经济发展后劲不足；资金、土地等发展要素制约较多，环境保护和节能减排任务艰巨；涉及群众切身利益的问题仍然突出，在房屋征收、土地征用、回迁安置、就医就学、道路交通等方面还存在群众不满意的地方；政府自身建设还需进一步加强，一些部门和机关干部事业心、责任感不强，主动性、创造性工作不够，办事效率低、服务意识差等现象仍然存在。对这些矛盾和问题，我们必须以对鞍山发展高度负责的精神，采取更加有力的措施，认真加以解决，决不辜负全市人民的期望。

二、2012年政府工作任务

2012年，是“十二五”承上启下的重要一年。新的一年，我们面临的经济形势更加错综复杂，面临的挑战异常严峻。特别是支撑我市经济发展的钢铁和房地产两大主导产业下滑趋势凸显，保增长的压力加大、难度加大。因此，我们一定要保持清醒头脑，增强忧患意识、危机意识，充分估计形势的复杂性和严峻性，积极应对，攻坚克难，逆势而上，确保完成全年工作任务。

今年政府工作的总体要求是：高举中国特色社会主义伟大旗帜，以邓小平理论和“三个代表”重要思想为指导，深入贯彻落实科学发展观，认真贯彻落实市第十一次党代会和市委十一届二次全会精神，深刻把握“稳中求进，稳中求快”总基调，以科学发展、创新发展、和谐发展为主题，以经济转型、社会转型、体制转型为主线，以促发展、保民生、优环境为主要任务，着力推进经济平稳较快发展，着力保障和改善民生，着力打造优良的投资和人居环境，加快建设富庶文明幸福新鞍山。主要预期目标是：地区生产总值增长12%，全社会固定资产投资增长20%，地方财政一般预算收入增长15%，城市居民人均可支配收入增长12%，农民人均纯收入增长11%。

（一）加快发展，全面提升经济综合实力

坚持把发展作为第一要务，以存量吸增量，以增量调结构，以创新促升级，以开放促发展，改造提升传统产业，培育发展战略性新兴产业，全面优化产业结构。

1.加快工业经济发展

牢固树立工业强市理念。工业是立市之本，强市之基，是实现鞍山平稳较快发展的重要支撑。要深入实施“工业五项工程”，大力发展五大工业主导产业，举全市之力建设激光产业基地。重点支持骨干企业发展，扶持销售收入超10亿元企业做大做强，培育一批销售收入10亿元、50亿元、100亿元以上工业企业。全力跟踪重点项目，积极推进五矿投资300亿元建设装备制造产业基地项目，年内开工建设亿元以上工业项目200个，全年工业增加值增长15%以上。

全力支持鞍钢等中省直企业发展。坚定不移地支持鞍钢发展，与鞍钢同呼吸共患难，共渡难关，为鞍钢打造最具国际竞争力的特大型跨国集团创造条件。深化地企融合，支持鞍钢并购地方企业，拉动地方企业做大做强。继续支持中国三冶、中冶焦耐、中冶北方、中钢热能等中省直企业优化产业结构，加快发展总部经济。

大力扶持民营企业发展。鼓励和支持中小企业通过“上船重组、上市融资、上档改造”发展壮大。全年并购国内外科技型企业10户，引进海外研发团队10个。强化政策支持，拓宽融资渠道，推进技术创新，优化服务环境，促进民营经济快速发展，全年民营经济增加值增长18%。

2.加快现代服务业发展

优化服务业发展布局。围绕打造商务集聚区、旅游集聚区和物流集聚区，重点推进汤岗子新城、达道湾新城、铁东南部新区、铁西高铁新区、千山文化旅游产业带、万水河景观带、建国大道服务业集聚带建设，形成“两城”、“两区”、“三带”服务业发展新格局。年内开工建设亿元以上服务业项目215个，社会消费品零售额增长16%。

大力发展金融业。鼓励金融机构扩大信贷投放，特别是要积极争取国开行和农发行的信贷支持，力争新增贷款230亿元。鼓励企业进入资本市场，年内实现2户企业上市。拓宽融资渠道，大力发展投资基金公司、村镇银行、担保公司和小额贷款公司，积极开展金融租赁融资业务，直接融资比率超过全省平均水平。

保持房地产业健康发展。认真落实中央调控措施，全面发展工业地产、商业地产、旅游地产和住宅地产，房地产开发投资和销售额增长15%以上。

3.加快县域经济发展

推进农业产业化。重点扶持农业产业化龙头项目，大力推进海城大蒜、台安鸭鹅、岫岩蘑菇农业主导产业集群发展。重点推进投资10亿元以上的农产品深加工项目。发展壮大农副产品专业批发市场，建立现代流通体系，实施农超对接，降低农产品流通成本，促进农民增收。

推进工业产业集群建设。突出主导产业发展，加速打造超千亿元的海城菱镁新材料产业集群，超百亿元的台安石油化工、岫岩矿产品深加工产业集群，全年县域工业开工千万元以上项目400个。

推进县域服务业发展。加快商贸和旅游集聚区建设，重点推进西柳商贸城、南台皮革城、玉皇山观光带、辽河生态旅游观光带、孤山仙人洞遗址等项目建设。

推进县城和新城新市镇建设。科学规划县城，高标准建设县城。按照政府主导、市场化运作原则，积极引进战略投资者，加快海西、腾鳌新城和牛庄、黄沙、雅河新市镇建设。坚持依法依规、节约集约用地，切实保护被征地农民合法权益。

4.全面扩大改革开放

加大深化改革力度。进一步下放管理权限，赋予高新区、鞍山经济开发区市级管理权限，赋予城区县级管理权限，力争鞍山经济开发区进入国家级开发区。

加大招商引资力度。抓住国内外产业转移的有利时机，重点面向欧美和香港、长三角、珠三角等资本密集区及行业龙头企业、中央大企业、世界500强企业招商。突出主题概念招商、产业集群招商，依托鞍钢资源对外招商，鼓励地方企业重组招商，重点引进具有牵动作用、支撑未来经济发展的大项目。全年实际利用外商直接投资增长15%以上，引进域外资金增长25%以上。

加大对上争取资金和政策支持力度。及时把握国家政策和专项资金扶持方向，重点围绕保障性住房、农田水利、扶贫开发、战略性新兴产业和节能环保等国家支持领域，积极争取国家和省资金、政策支持，全年对上争取资金力争150亿元，比上年增长50%。

5.全力抓好项目建设

实施项目牵动战略。项目建设既是增强发展后劲的长远需要，也是保增长的现实支撑，也是今年工作的重中之重。市委、市政府确定今年为项目建设年，要举全市之力推进项目建设。在项目建设中，要坚持工业、农业、商业、旅游、地产项目一起上，大项目、小项目一起上，形成项目建设“铺天盖地”的局面。

着力推进重大项目开工建设。全年开工建设亿元以上项目480个，总投资5926亿元。重点推进港中旅温泉城等16个投资百亿元以上项目建设，重点推进辽宁缘泰20万吨丁辛醇等35个投资50亿元以上项目建设，重点推进中南世贸中心等125个投资10亿元以上项目建设。要着力解决项目落地和建设中的土地、资金、环评、基础设施配套等问题，千方百计变“纸上项目”为“落地项目”、“围墙项目”为“建设项目”、“空巢项目”为“达产项目”，以大项目建设推动经济大发展。

（二）以人为本，全面推进社会事业发展

坚持执政为民，把改善民生和发展社会事业摆到更加突出的位置，让全市人民共享改革发展成果。

1.保障和改善民生，促进社会和谐发展

全力稳定和扩大就业。实施积极的就业政策，促进大学毕业生、退役士兵、农民工、新生劳动力和就业困难群体就业。全年新增实名制就业10万人，城镇登记失业率控制在3%以内，零就业家庭保持动态为零。

继续完善社会保障体系。加大社保扩面和征缴力度，推进城镇居民社会养老保险和农村新型社会养老保险全覆盖，确保企业离退休人员基本养老金按时调增和足额发放。全面实施城镇基本医疗保险市级统筹，促进城乡医疗保障均等化。将事业单位纳入工伤保险覆盖范围。强化低保动态管理，提高城乡低保户和五保户保障标准。加快养老家园建设，大力推进社会养老事业和慈善事业发展。

切实改善群众生活条件。继续为城乡群众办实事。大力推进保障性住房建设和农村危房改造。多渠道筹集资金，加大回迁房建设力度，让动迁居民尽早回迁。强化价格监控，适时增加困难群体生活补贴。完善城乡救助体系，对城乡困难家庭子女全部资助就学，对农村困难家庭常年病人全部实施托管，对城乡孤残儿童全部实施免费托管和康复治疗，切实解决贫困人口和弱势群体生产生活困难问题。

加强社会管理。强化群众来信来访工作，妥善处理社会矛盾，全力解决群众合理诉求，切实维护群众合法权益。强化社会治安综合治理，全面完善社会治安防控体系，不断增强人民群众安全感。强化食品药品监管，严厉打击制售假冒伪劣食品和药品等违法犯罪行为，保证人民群众饮食和用药安全。加强交通秩序整治，切实改善交通环境。加强安全生产和消防安全，坚决遏制重特大安全事故发生。

2.统筹兼顾，协调发展各项社会事业

优先发展教育事业。建立公立幼儿教育集团，鼓励社会资本兴办幼儿教育，切实解决“入园难、入园贵”问题。改善办学环境，新建10所中小学校，新建和改造5所高中。积极创建国家中等职业教育改革发展示范校，把市职教城打造成为全国职业教育示范基地。积极为辽宁科技大学、鞍山师范学院发展及重点学科建设创造良好条件。

深化医药卫生体制改革。深入推进公立医院改革，创新公立医院体制机制，鼓励和引进社会资本兴办医疗机构，努力走出一条可持续、可借鉴、有示范作用的新路子。健全公共卫生服务体系，提升基本医疗服务能力，解决“看病难、看病贵、看不好病”的问题。建成公共卫生中心，开工建设中国医大鞍山医院、医药大市场等重点项目。

加快建设文化强市。坚持一手抓文化事业，一手抓文化产业。加快推进胜利会堂改造和社区文化中心等文化设施建设，不断丰富人民群众文化生活。加快文化产业建设，推动文化产业成为我市经济支柱性产业。完成市奥体中心一场三馆和市民健身中心工程建设，做好第十二届全运会我市承办项目筹备工作。

继续做好人口和计划生育、民族、宗教、外事、侨务、老龄、残联、妇女儿童、优抚安置、防灾减灾、国家安全和双拥共建等各项工作。

（三）优化环境，全面提升城市可持续发展能力

坚持统筹协调发展，加强城市建设与管理，不断改善生态环境，强化公共服务功能，努力建设宜居宜业的现代化城市。

1.优化城市环境

加强城市规划。加快城市总体规划修编，进一步完善新城规划、新区规划、分区规划和专项规划。严格规划审批和监管，严格按照规划建设城市。

加强城市建设。全面推进汤岗子新城、达道湾新城、铁东南部新区、铁西高铁新区基础设施建设。推进凤凰湖、杨柳湖、运粮湖、静湖等滨水公园建设，开工建设营城子公园和市动物园改造工程。提升改造羊耳峪垃圾处理厂，启动建设垃圾焚烧处理厂。加快推进御泉路、解放大道、建设大道以及沈西工业走廊出海通道鞍山段、鞍台高速公路、盘（海）营客运专线等重点交通工程建设。

加强城市管理。按照规范化、系统化、精细化原则，全面加强城市管理。充分发挥城区政府职能作用，推进城市管理重心下移。继续开展市容市貌“十项综合整治”行动，严厉打击破坏、盗窃和占用市政基础设施行为，让鞍山市民生活在更加舒适的城市环境中。

2.优化生态环境

实施青山工程。以我市被确定为全省唯一的青山工程示范市为契机，强化“还绿”、“造绿”、“护绿”、“管绿”，积极创建国家森林城市。综合整治矿山1万亩，力争3年全部恢复山体生态；植树造林29万亩，完成小开荒还林2万亩、坡耕地和沙化地还林4万亩；严格保护城市山体环境和山林资源，建设封山禁牧围栏145公里；强化青山保护和管理，力争全市森林覆盖率达到48%以上。

实施碧水工程。推进大孤山、宁远、达道湾等7座污水处理厂建设和提标改造，推进污泥处理厂建设，全市形成63.5万吨污水日处理能力。推进南沙河、杨柳河、海城河、五道河河道综合整治工程建设，推进大麦科人工湿地建设和大张、红庙子、京沈桥湿地综合整治，切实保护三岔河湿地自然保护区，完成辽浑太三大河鞍山段水污染治理任务，逐步恢复河水自净能力。

实施蓝天工程。重点推进鞍钢东烧、后英钢铁和恒盛铸业等7台烧结机脱硫设施建设，推进燃煤锅炉脱硝和低氮燃烧设施建设。大力发展集中供热，积极推进大唐2台35万千瓦和国电鞍山2台30万千瓦热电联产项目。积极推广清洁能源，引进“西气东输”天然气和大连液化天然气。综合整治建筑施工、矿山排岩等扬尘污染，全面整治机动车尾气污染。全面改善城市空气环境质量，力争城区85%以上天数的空气环境质量达到国家二级标准。

“三大工程”既是生态环境工程，也是重点民生工程。我们要坚定不移地全面推进，让鞍山的山更绿、水更清、天更蓝、环境更美。

3.优化服务环境

认真为民做事。始终把关注民生、改善民生作为政府工作的出发点和落脚点，一心一意想着群众、一心一意为了群众，切实解决人民群众最关心、最直接、最现实的利益问题，让改革发展的成果最大限度地惠及广大人民群众。

严格依法办事。坚持重大事项向市人大及其常委会报告制度，积极支持市政协履行政治协商和参政议政职能，自觉接受市人大及其常委会的法律监督、工作监督和市政协的民主监督。认真办理人大议案、代表建议和政协提案。推进科学民主决策，坚持重大事项集体讨论、专家论证、社会公示和公开听证制度，提高决策透明度和公共参与度。推进政务公开，减少审批事项，简化审批手续，提高办事效率，营造优良的投资环境。

务实创新成事。坚持求真务实，真抓实干，说实话，办实事，求实效。强化目标责任，强化督查督办，强化绩效考评，形成权责一致、分工合理、执行顺畅、监督有力的行政管理体制。用创新思维增强破解难题能力，着力解决制约影响改革发展稳定的突出问题。

清正廉洁干事。坚持从严治政，强化权力监管，推进惩防体系建设，加强公共资源交易系统建设，加大重点领域行政监察和审计监督力度，确保权力在阳光下运行。牢固树立过紧日子思想，开源节流，勤俭节约，把有限的资金用在促进发展上，用在改善民生上。坚决纠正损害人民群众利益的不正之风，严肃查处各类违法违纪案件，决不让腐败问题影响鞍山的整体形象，决不让腐败问题影响鞍山的振兴大业。

各位代表，我们面临的任务艰巨而繁重，我们肩负的责任重大而光荣。让我们在市委的领导下，在市人大、市政协监督支持下，团结带领全市各族人民，锐意进取，扎实工作，以更顽强的斗志、更务实的作风、更有力的措施，全力推进鞍山科学发展、创新发展、和谐发展，为建设富庶文明幸福新鞍山作出新的更大的贡献！
